# Association of lipoprotein(a) with intrinsic and on-clopidogrel platelet reactivity

**DOI:** 10.1007/s11239-021-02515-2

**Published:** 2021-07-02

**Authors:** Alexander Kille, Thomas Nührenberg, Kilian Franke, Christian M. Valina, Gregor Leibundgut, Sotirios Tsimikas, Franz-Josef Neumann, Willibald Hochholzer

**Affiliations:** 1 Department of Cardiology and Angiology II, Medical Center, University of Freiburg, University Heart Center Freiburg-Bad Krozingen, Suedring 15, 79189 Bad Krozingen, Germany; 2 Cantonal Hospital Baselland, Liestal, Switzerland; 3 Sulpizio Cardiovascular Center, Division of Cardiovascular Medicine, University of California San Diego, San Diego, USA

**Keywords:** Lipoprotein(a), Platelet reactivity, Dual antiplatelet therapy (DAPT), Coronary arterial disease, Percutaneous coronary intervention, Riscfactor

## Abstract

Lipoprotein(a) [Lp(a)] is an independent, genetically determined, and causal risk factor for cardiovascular disease. Laboratory data have suggested an interaction of Lp(a) with platelet function, potentially caused by its interaction with platelet receptors. So far, the potential association of Lp(a) with platelet activation and reactivity has not been proven in larger clinical cohorts. This study analyzed intrinsic platelet reactivity before loading with clopidogrel 600 mg and on-treatment platelet reactivity tested 24 h following loading in patients undergoing elective coronary angiography. Platelet reactivity was tested by optical aggregometry following stimulation with collagen or adenosine diphosphate as well as by flow cytometry. Lp(a) levels were directly measured in all patients from fresh samples. The present analysis included 1912 patients. Lp(a) levels ranged between 0 and 332 mg/dl. There was a significant association of rising levels of Lp(a) with a higher prevalence of a history of ischemic heart disease (p < 0.001) and more extensive coronary artery disease (p = 0.001). Results for intrinsic (p = 0.80) and on-clopidogrel platelet reactivity (p = 0.81) did not differ between quartiles of Lp(a) levels. Flow cytometry analyses of expression of different platelet surface proteins (CD41, CD62P or PAC-1) confirmed these findings. Correlation analyses of levels of Lp(a) with any of the tested platelet activation markers did not show any correlation. The present data do not support the hypothesis of an interaction of Lp(a) with platelet reactivity.

**Supplementary Information:**

The online version contains supplementary material available at 10.1007/s11239-021-02515-2.

## Highlights

- Lipoprotein(a) is as risk factor for coronary events.
- Laboratory data have suggested an interaction of Lp(a) with platelet function.
- The present data do not support the hypothesis of an interaction of Lp(a) with intrinsic or on dual antiplatelet therapy platelet reactivity but confirms the importance of Lp(a) as risk factor for coronary events.
- These findings might be important to define the safety of evolving therapeutic options for lowering Lp(a).

## Introduction

Lipoprotein(a) [Lp(a)] is an independent and causal risk factor for premature coronary heart disease, myocardial infarction (MI), atherosclerosis, aortic valve stenosis, and stroke [1–4]. Lp(a) is a LDL-like particle, which consists of an apolipoprotein B-100 disulfide-linked to an apolipoprotein(a) [Apo(a)] [5]. Levels of Lp(a) are primarily genetically determined, have limited variability within a pre-defined range among individuals, and show a wide variance among different populations [2, 6]. Lp(a) appears to be an attractive target for reduction of cardiovascular events since retrospective analyses have suggested that lowering of levels of Lp(a) appears to be associated with a reduced cardiovascular risk [4]. In particular, antisense oligonucleotide therapy seems to be a promising way to lower levels of Lp(a) [7, 8].

**Fig. 1:**
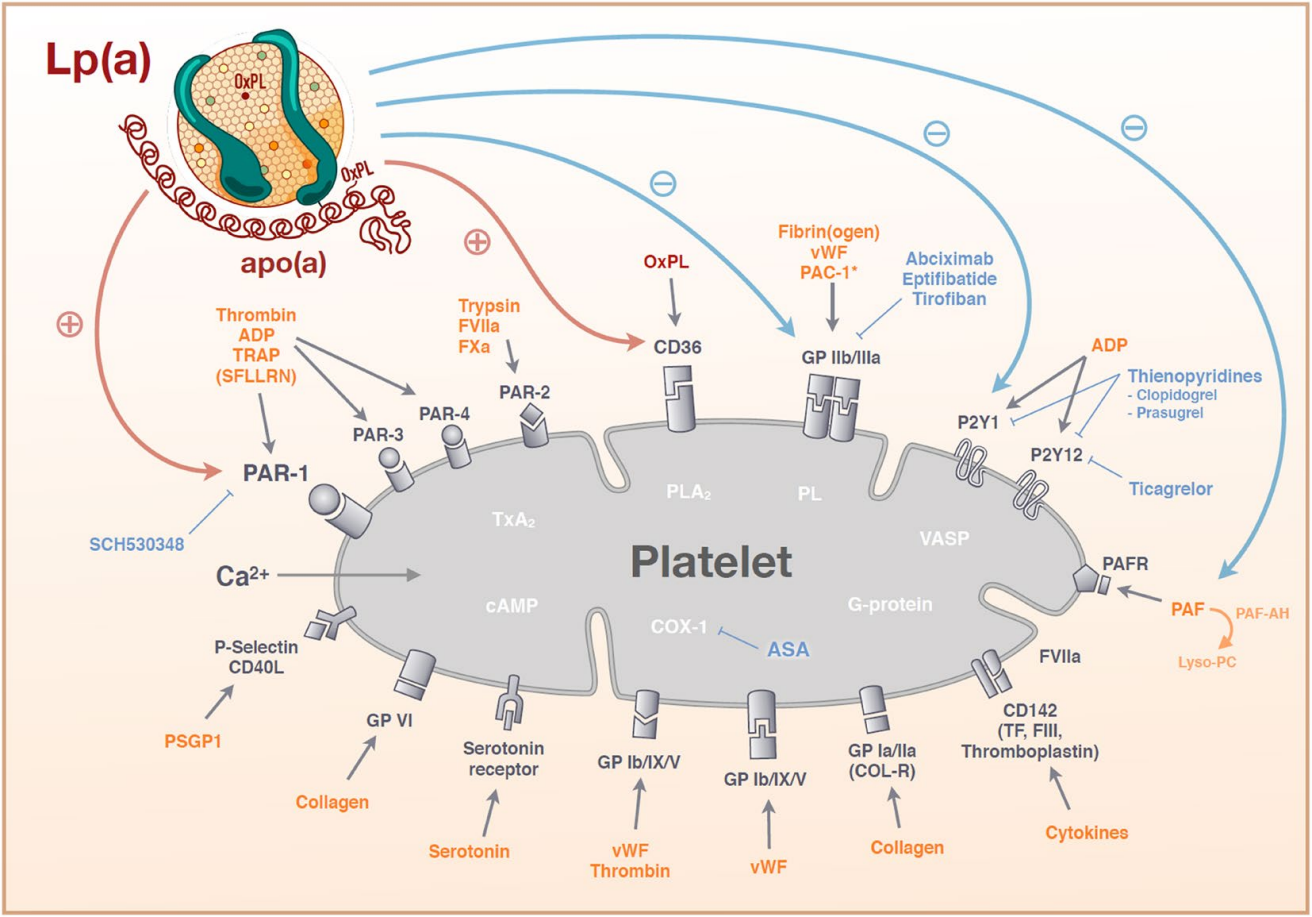
Potential pathways of interaction of LP(a) with platelet function. Shown is an overview of important platelet receptors with its ligands. Potential receptors by which Lp(a) as ligand could activate (red arrows) or inhibit (blue arrows) platelet aggregation are presented. *ADP* adenosine diphosphate, *ASA* acetylsalicylic acid, *cAMP* Cyclic adenosine monophosphate, *COX-1* cyclooxygenase-1, *Lyso-PC* Lysophosphatidylcholines, *OxPL* oxidized phospholipids, *PAC-1* procaspase-activating compound 1, *PAF* platelet-activating factor, *PLA*_*2*_ Phospholipases A2, *PSGP1* P-selectin glycoprotein ligand 1, *SCH530348* Vorapaxar, TRAP/SFLLRN thrombin receptor activating peptide, *TxA*_*2*_ Thromboxan-A2, *VASP* vasodilator-stimulated phosphoprotein, *vWF* von willebrand factor

The association of Lp(a) with potential anti-fibrinolytic activity is well known, yet the clinical relevance of this has been recently questioned by studies showing no association of genetically determined Lp(a) levels with deep venous thromboses [9]. In addition, several studies have indicated that there might be a potential interaction of Lp(a) with the plasma coagulation cascade and platelet function through its apo(a) component and content of oxidized phospholipids. For example, Lp(a) may activate platelets through the surface receptors PAR-1 and CD36 and in contrast, may prevent latent activation/aggregation through the receptors GPIIb/IIIa and P2Y1 as well as by interacting with platelet activating factor (Fig. 1) [10–22]. The net effect of these divergent interactions cannot be easily determined in vitro. Therefore, patient cohorts on therapies affecting these pathways are required to assess their clinical relevance. Given the novel therapeutic options for reducing levels of Lp(a), it appears of importance to evaluate this potential interaction to define the safety of lowering Lp(a). Thus, the present study sought to investigate the potential association of Lp(a) levels with platelet activation and on-treatment platelet reactivity in a large clinical cohort.

## Material and methods

### Study population

The present study is a secondary analysis of the prospective EXCELSIOR study (Impact of Extent of Clopidogrel-Induced Platelet Inhibition during Elective Stent Implantation on Clinical Event Rate; ClinicalTrials.gov Identifier: NCT00457236), which investigated platelet reactivity in patients undergoing elective coronary angiography as potential candidates for percutaneous coronary intervention (PCI) [23]. The study was approved by the ethics committee of the University of Freiburg, Germany. Written informed consent was given by all participants.

All patients received a pre-treatment with 600 mg of clopidogrel and were on aspirin before coronary angiography. Patients not on aspirin ≥ 100 mg daily for five days bevor enrolment received an oral loading dose of 400 mg (24.7% of patients, given at mean 3.1 h bevor coronary angiography). Key exclusion criteria were chronic treatment with clopidogrel or ticlopidine or current oral anticoagulation, contraindication to aspirin, clopidogrel or heparin as well as active cancer or terminal renal failure. PCI was performed as previously described [23, 24] and was timed according to the routine schedule of the catheterization laboratory. All patients received a maintenance therapy with clopidogrel (75 mg daily) if coronary intervention was performed together with aspirin (≥ 100 mg) lifelong. Patients were contacted by telephone up to 10 years following enrolment to obtain a complete long-term follow-up. Key clinical endpoints were death of any cause and MI according to the Universal Definition of Myocardial Infarction.

### Platelet function studies

Blood samples were drawn before loading with clopidogrel to test intrinsic platelet reactivity and at day 1 following loading 2–4 h after intake of the first maintenance dose of clopidogrel. Tubes containing 3.2% sodium-citrate (Sarstedt, Nuembrecht, Germany) were used for blood drawing and processed within 1 h after. Turbidimetric aggregometry using a four-channel Bio/Data PAP4 aggregometer (Mölab, Langenfeld, Germany) was used to test platelet aggregation as marker for platelet reactivity as previously described [23, 25]. Platelet-rich plasma was prepared by centrifugation of citrated venous blood at 750 g for 2 min and adjusted to 275–325 × 109 thrombocytes/l by dilution with platelet-poor plasma from the same patient. To induce aggregation Adenosine diphosphate (ADP) (Sigma-Aldrich, Munich, Germany), collagen (Nycomed Pharma, Unterschleissheim, Germany), or arachidonic acid (Mölab, Hilden, Germany) were added. In Platelet-rich plasma light transmission aggregometry was performed and was determined 5 min after adding ADP at a final concentration of 5 µM or collagen 2.5 mg/L or arachidonic acid 500 mg/L. Results were expressed as percentage of maximal light transmission using platelet-poor plasma from the same patient as reference (= 100% aggregation). The used optical aggregometry assay has a coefficient of variation of 6.1% [23, 24]. Expression of P-selectin and activated GP IIb/IIIa were determined by triple color flow cytometry (FACS) after staining of the platelets with FITC-tagged PAC-1 (Becton Dickinson, Heidelberg, Germany), PE-tagged CD62P and PC7-tagged CD41 (Beckman Coulter, Krefeld, Germany) monoclonal antibodies and incubation with 20 μM ADP as described previously [25].

### Lp(a) measurement

At hospital admission, levels of Lp(a) were measured as a routine laboratory parameter using an immunoturbidimetric assay (Tina-quant®, Roche, Bale, Switzerland) on a Hitachi-Modular system.

### Statistical methods

For all analyses, continuous variables are presented as median ± interquartile range and discrete variables are reported as counts (percentages). We used the χ^2^-test to test for differences between groups of discrete variables and the Kruskal–Wallis test for non-Gaussian variables. A p value < 0.05 was regarded as significant. To analyze the impact of the Lp(a)-levels on platelet reactivity, patients were stratified into quartiles of Lp(a) levels in a similar fashion to previous studies. In addition, we investigated the influence of Lp (a) as a continuous variable using logistic regression and for correlation Spearman-Rho. Follow-up data were tested by cox regression analysis. The SPSS software package, version 25, was used for all analyses (IBM Corporation, Armonk, NY, USA). Statistical graphs were designed with GraphPad PRISM 8 (GraphPad Software, San Diego, CA, USA).

## Results

**Fig. 2:**
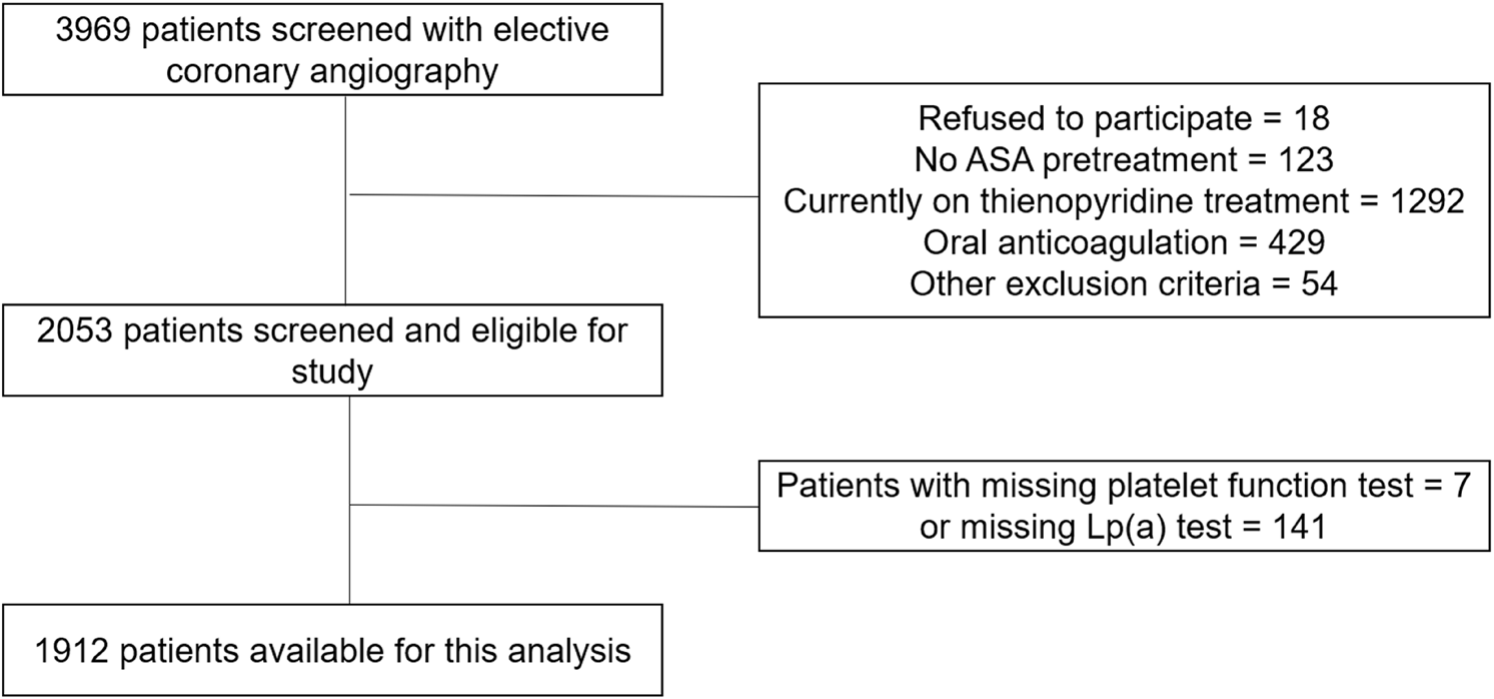
Study flow. Shown is the study flow regarding to the inclusion/ exclusion criteria. *ASA* acetylsalicylic acid

In total, 3696 patients were screened and 1912 patients with available platelet function and Lp(a) test results could be included in this analysis (Fig. 2). The median age of the patients was 66 [59–72] years and 67.9% were male. Levels of Lp(a) ranged between 0 and 332 mg/dl. There was no significant difference between quartiles of Lp(a) levels for age, left ventricular function or several cardiovascular risk factors such as arterial hypertension, diabetes mellitus, or obesity. Low-density lipoprotein cholesterol did not differ between quartiles whereas high-density lipoprotein cholesterol demonstrated significant but numerically small differences between quartiles.

The prevalence of hypercholesterolemia, a positive family history for cardiovascular disease or a previous MI, PCI or coronary artery bypass grafting increased with rising levels of Lp(a) (Table 1). A similar association was found on coronary angiography: the severity of coronary heart disease was increasing with rising levels of Lp(a). Almost 70% of patients in the highest quartile of Lp(a) levels had a coronary stenosis ≥ 75% or a stenosis ≥ 50% of the left main coronary artery. This led to an indication for PCI in more than 57% of patients in the highest quartile of Lp(a). The association of levels of Lp(a) and cardiovascular disease was also confirmed during clinical follow-up of 5.6 years. During this period, 222 (11.6%) patients died and MI occurred in 103 (5.4%) patients. Levels of Lp(a) were significantly associated with the risk of death or MI (hazard ratio 1.005; 95%-confidence interval 1.001–1.008; p = 0.01).

**Table 1:** Baseline characteristics

	Whole population n = 1912	Lp(a) < 9 mg/dl n = 494	Lp(a) 9-20 mg/dl n = 481	Lp(a) 21-58 mg/dl n = 465	Lp(a) > 58 mg/dl n = 472	p value
Age (years)	66 [59–72]	66 [59–73]	65 [59–72]	66 [61–72]	66 [59–72]	0.33
Male sex	1298 (67.9%)	342 (69.2%)	328 (68.2%)	313 (67.3%)	315(66.7%)	0.85
Hypertension	1503 (78.6%)	383 (77.5%)	373 (77.5%)	376 (80.9%)	371 (78.6)	0.56
Diabetes mellitus	404 (21.1%)	112 (22.7%)	111 (23.1%)	94 (20.2%)	87 (18.4%)	0.25
Obesity	594 (31.1%)	169 (34.2%)	148 (30.8%)	137 (29.5%)	140 (29.7%)	0.35
History of smoking	656 (34.3%)	173 (35.0%)	171 (36.0%)	142 (30.5%)	168 (35.6%)	0.23
Family history for CAD	596 (31.2%)	128 (25.9%)	154 (32.0%)	148 (31.8%)	166 (35.2%)	0.02
Hypercholesterolemia	1674 (87.6%)	426 (86.2%)	416 (86.5%)	400 (86.0%)	432 (91.5%)	< 0.001
Statin therapy	974 (50.9%)	221 (44.7%)	221 (45.9%)	235 (50.5%)	297 (62.9%)	< 0.001
Previous PCI	579 (30.3%)	135 (27.3%)	133 (27.7%)	129 (27.7%)	182 (38.6%)	< 0.001
Previous CABG	212 (11.1%)	38 (7.7%)	43 (8.9%)	48 (10.3%)	83 (17.6%)	< 0.001
Previous myocardial infarction	416 (21.8%)	31 (18.4%)	96 (20.0%)	90 (19.4%)	139 (29.4%)	< 0.001
Severity of coronary obstruction						
< 20%	287 (15.0%)	78 (15.8%)	78 (16.2%)	87 (18.7%)	44 (9.3%)	0.001
20–49%	327 (17.1%)	95 (19.2%)	88 (18.3%)	76 (16.3%)	68 (14.4%)	
50–74%	155 (8.1%)	37 (7.5%)	42 (8.7%)	38 (8.2%)	38 (8.1%)	
≥ 75% o. LM ≥ 50%	1143 (59.8%)	284 (57.5%)	273 (56.8%)	264 (56.8%)	322 (68.2%)	
Indication for PCI	936 (49.1%)	238 (48.2%)	224 (46.6%)	204 (43.9%)	272 (57.6%)	< 0.001
HDL-Chol. (mg/dl)	54 [45–65]	53 [45–64]	53 [44–63]	55 [46–66]	55 [47–66]	0.02
LDL-Chol. (mg/dl)	126 [102–153]	123 [100–151]	126 [102–154]	127 [103–153]	127 [104–153]	0.31
Normal LV-Function	1293 (67.6%)	338 (68.4%)	337 (70.1%)	321 (69.0%)	297 (62.9%)	0.12

*CAD* coronary arterial disease, *PCI* percutaneous coronary intervention, *CABG* coronary artery bypass grafting, *LM* left main, *HDL* = high density lipoprotein, *LDL* low density lipoprotein, *LV* left ventricle

**Fig. 3:**
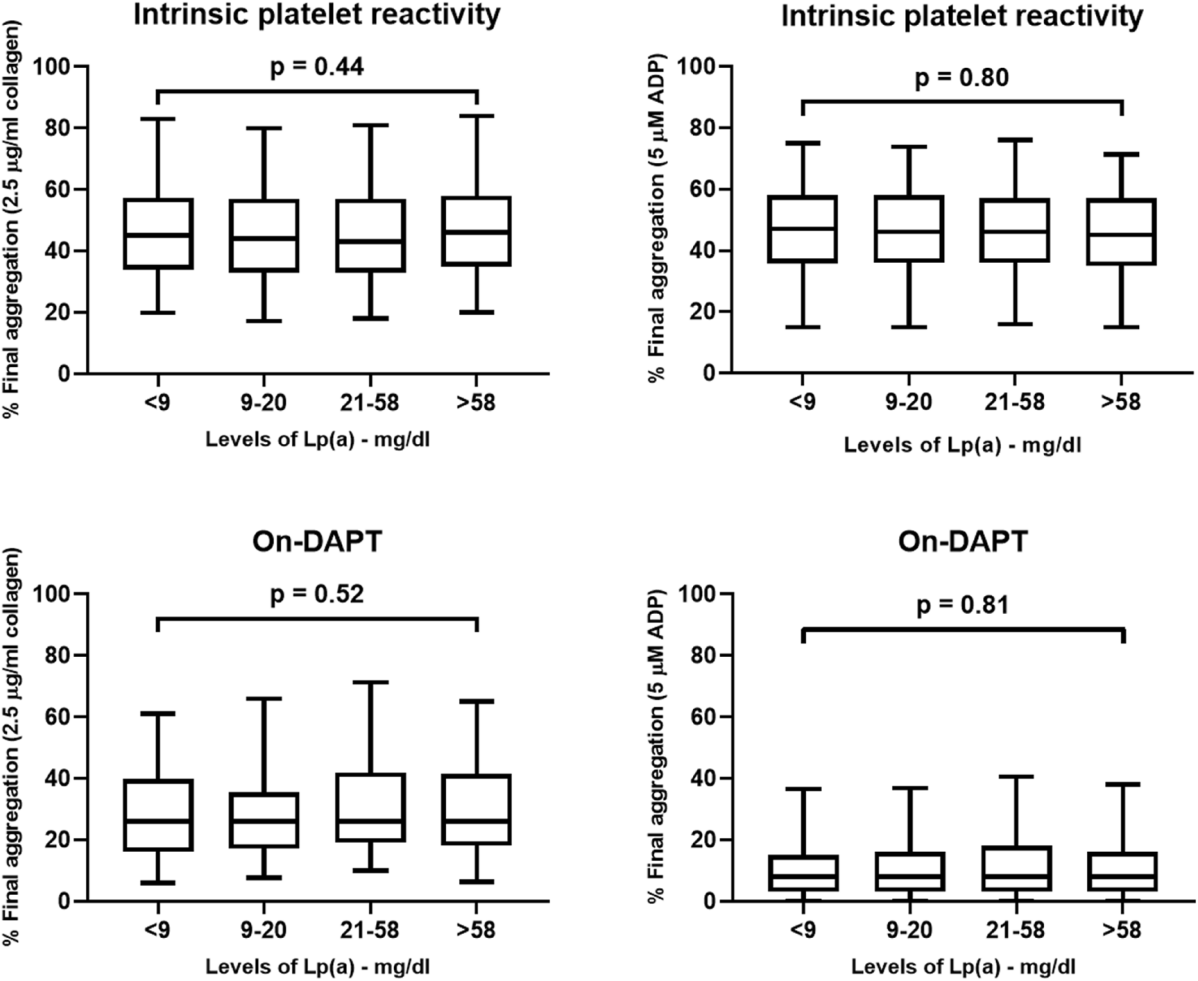
Light transmission aggregometry stratified according to quartiles of LP(a). Shown is the intrinsic and on-dual antiplatelet therapy (DAPT) platelet reactivity tested 24 h following loading with clopidogrel

**Fig. 4:**
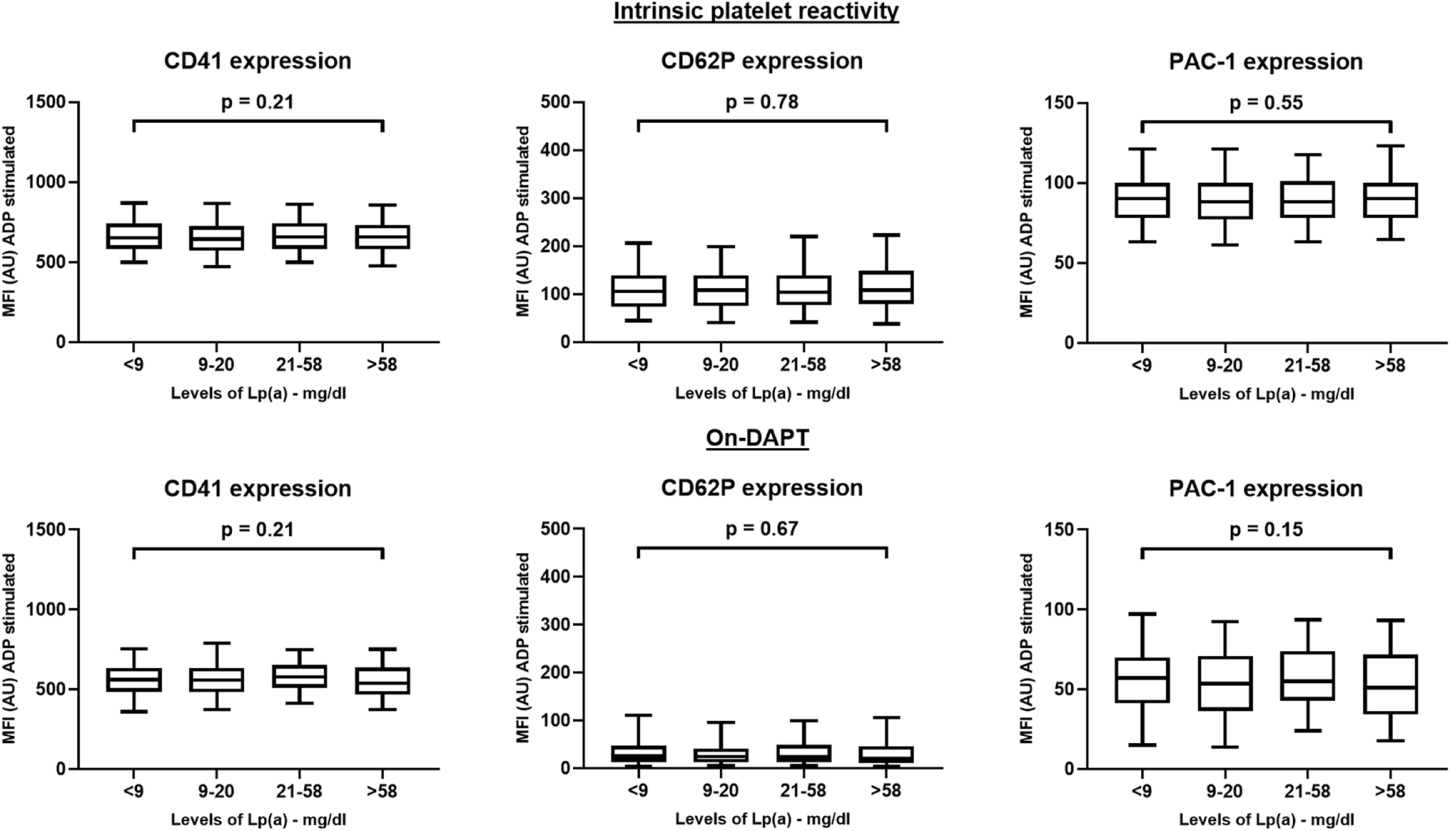
Surface protein expression stratified according to quartiles of LP(a). Shown is the intrinsic and on- dual antiplatelet therapy (DAPT) platelet reactivity tested 24 h following loading with clopidogrel

Platelet reactivity as tested with light transmission aggregometry did not show any differences between quartiles of Lp(a). Following stimulation with collagen or ADP, there was no significant and almost no numerical difference between quartiles for both, intrinsic and on-clopidogrel platelet reactivity (Fig. 3). Platelet reactivity following stimulation with arachidonic acid to test the effect of aspirin did also not differ between quartiles (p = 0.44). Surface protein expression of CD41, CD62P and PAC-1 following stimulation with ADP as assessed by FACS demonstrated similar results between the four quartiles of Lp(a) before as well as after loading with clopidogrel (Fig. 4). Furthermore, there was no correlation between levels of Lp(a) and results of platelet function studies (Table 2). However, platelet function correlated well with previously described predictors of platelet reactivity such as age, body mass index, diabetes, or platelet count.

**Table 2:** Correlation of Lp(a) and clinical variables with platelet reactivity

A		Intrinsic platelet reactivity			On-DAPT platelet reactivity	
	Stimulating reagent	Arachidonic acid (500 mg/L)	Collagen (2.5 µg/ml)	ADP (5 µM)	Collagen (2.5 µg/ml)	ADP (5 µM)
Lp(a)	Correlation coefficient	0.018	0.000	− 0.024	0.042	0.009
	p value	0.423	0.997	0.291	0.261	0.818
Age	Correlation coefficient	0.020	0.047	0.137	0.087	0.133
	p value	< 0.001	0.041	< 0.001	0.018	< 0.001
Body mass index	Correlation coefficient	0.020	0.031	0.028	0.035	0.091
	p value	0.382	0.179	0.226	0.341	0.014
Diabetes	Correlation coefficient	0.037	0.036	0.025	0.117	0.128
	p value	0.111	0.111	0.279	0.002	0.001
History of smoking	Correlation coefficient	− 0.058	− 0.036	− 0.057	0.061	0.042
	p value	0.011	0.117	0.013	0.099	0.259
Creatinine level	Correlation coefficient	0.016	− 0.079	− 0.041	− 0.011	0.014
	p value	0.483	0.001	0.074	0.765	0.708
Platelet count	Correlation coefficient	− 0.035	− 0.056	− 0.074	− 0.119	− 0.114
	p value	0.129	0.014	0.001	0.001	0.002

B		Intrinsic platelet reactivity			On-DAPT platelet reactivity		
		CD41	CD62P	PAC-1	CD41	CD62P	PAC-1
Lp(a)	Correlation coefficient	0.001	0.020	0.005	0.005	− 0.027	− 0.032
	p value	0.957	0.409	0.827	0.903	0.491	0.426
Age	Correlation coefficient	− 0.058	0.062	0.006	− 0.038	− 0.003	0.043
	p value	0.017	0.011	0.808	0.342	0.938	0.274
Body mass index	Correlation coefficient	− 0.029	− 0.035	− 0.054	0.044	0.055	0.082
	p value	0.230	0.156	0.026	0.272	0.164	0.038
Diabetes	Correlation coefficient	− 0.005	0.011	− 0.056	0.013	0.079	0.069
	p value	0.848	0.664	0.022	0.747	0.046	0.080
History of smoking	Correlation coefficient	− 0.016	− 0.030	− 0.021	0.051	0.081	0.077
	p value	0.512	0.224	0.392	0.196	0.040	0.051
Creatinine level	Correlation coefficient	0.065	− 0.026	− 0.063	0.019	− 0.001	− 0.036
	p value	0.008	0.284	0.009	0.627	0.978	0.368
Platelet count	Correlation coefficient	− 0.135	− 0.031	− 0.110	− 0.152	− 0.035	− 0.127
	p value	< 0.001	0.211	< 0.001	< 0.001	0.380	0.001

Correlation of Lp(a) and clinical variables with platelet reactivity measured by light transmission aggregometry (A) and with surface protein expression following stimulation with ADP (B). Correlation coefficient and p value by Spearman correlation. Surface protein expression was measured under stimulation by ADP. *DAPT* dual antiplatelet therapy

## Discussion

The present study investigated the clinical significance of the proposed interaction of levels of Lp(a) with platelet reactivity. The key finding of the present study is that in contrast to previous in vitro findings, the present data from a large and well powered cohort do not indicate any association of Lp(a) and in-vivo platelet function in patients on aspirin or on dual antiplatelet therapy. Results of flow cytometry analyzing surface protein expression on platelets confirmed these results. These findings, along with similar data from coagulation studies [26] showing no effect on fibrinolysis parameters with potent Lp(a) lowering treatment, suggest that the risk for coronary vascular disease associated with levels of Lp(a) may be primarily mediated by pro-atherogenic and pro-inflammatory effects via its oxidized phospholipids rather [27] than by anti-fibrinolytic or anti-platelet effects. These findings may have implications in the mechanism of any potential benefit in an ongoing trial testing the “Lp(a) hypothesis” with Lp(a) lowering by an antisense oligonucleotide (ClinicalTrials.gov Identifier: NCT04023552).

Similar results were seen in a recently published small in-vivo study that did not find any difference between intrinsic platelet reactivity in patients with levels of Lp(a) < 50 mg/dl versus ≥ 50 mg/dl [28]. These findings are in contrast to the results of in-vitro studies showing multiple different pathways of interaction between Lp(a) and platelets (Fig. 1). Experimental studies have shown that Lp(a) binds specifically and reversibly to platelets [20–22]. It was suggested that there might be a specific receptor for Apo(a) on platelets which modulates platelet function [21]. After activation of platelets with ADP, thrombin or arachidonic acid, the affinity of platelets to Apo(a) did not change but the binding of Lp(a) increased by 2- to 10-fold [20, 21]. However, it is still not proven so far if Apo(a) can interact with the plasminogen receptor of platelets [14] given its similar structure as compared to plasminogen. Other data suggested that Lp(a) binds to the fibrinogen (GPIIb/IIIa) receptor of platelets independently of its Apo(a) subunit [22]. However, further studies demonstrated that the IIb subunit of the fibrinogen (GPIIb/IIIa) receptor is inactivated by Apo(a), which inhibits platelet aggregation [12, 18]. Apart from different ways of binding of Lp(a) to platelets, it was shown that Lp(a) has an impact on different pathways of platelets. Cyclic AMP levels in platelets are up- or downregulated dependent on levels of Lp(a) [11]. It was demonstrated that platelet activation by ADP is inhibited by Lp(a) whereas other studies could not confirm these findings [10, 15, 18, 19]. Platelet activation promoted by the platelet-activation factor is also inhibited by Lp(a) [13, 16]. Apo(a) can lead to a decreased thromboxane A_2_ production in platelets and serotonin release as well as inhibition of collagen induced aggregation [10, 19]. But there are not only inhibiting effects on platelets. Other studies indicated that platelet response to the thrombin receptor-activating peptide SFLLRN is enhanced by Apo(a) [15]. It was also shown that oxidized phospholipids, which are part of Lp(a), are playing a role in atherosclerosis in particular by interacting with the CD36 receptor on platelets [17]. Binding of Apo(a) appears also to promote aggregation of platelets as demonstrated by lower doses of arachidonic acid needed to induce aggregation following binding of Lp(a) [21].

Beside inhibiting and activating effects on platelets also binding of plasminogen and tissue-type plasminogen activator to platelets is inhibited by Lp(a) and it acts as a competitive inhibitor of plasminogen activation by tissue-type plasminogen activator on the surface of platelets. Fibrinolysis might be impaired by this mechanism [20].

Lowering Lp(a) might decrease the risk for major ischemic events. Currently, only niacin, lipid apheresis, and PCSK9-inhibitors [29, 30] have shown to reduce levels of Lp(a) whereas statin therapy has demonstrated no effect or an increase in levels of Lp(a) [31]. Antisense oligonucleotide therapy to lower Lp(a) [7, 8] seems to be a promising way but is not available for clinical use so far. However, this approach has reached phase III testing and might become available in the near future. Thus, the present data might help define the potential safety of this therapeutic approach by demonstrating that the previously suggested interaction of Lp(a) and platelet function does not appear to be of importance in the target population for Lp(a) lowering.

### Limitations

This analysis is retrospective with all adherent limitations. Since only patients admitted for elective coronary angiography were enrolled, the present data cannot be extrapolated to the overall population. The number of clinical endpoints within the analyzed follow-up was limited.

## Conclusions

The present data do not support the hypothesis of an interaction of Lp(a) with platelet reactivity but confirms the importance of Lp(a) as risk factor for coronary events. These findings might be important to define the safety of evolving therapeutic options for lowering Lp(a).

## Supplementary Information

Below is the link to the electronic supplementary material.

Supplementary file1 (DOCX 179 kb)

## Abbreviations

Lp(a): Lipoprotein(a)
MI: Myocardial infarction
Apo(a): Apolipoprotein(a)
PCI: Percutaneous coronary intervention
ADP: Adenosine diphosphate

## References

[1] Kamstrup PR, Tybjaerg-Hansen A, Steffensen R, Nordestgaard BG (2009). Genetically elevated lipoprotein(a) and increased risk of myocardial infarction. JAMA.

[2] Nordestgaard BG (2010). Lipoprotein(a) as a cardiovascular risk factor: current status. Eur Heart J.

[3] Nordestgaard BG, Langsted A (2016). Lipoprotein (a) as a cause of cardiovascular disease: insights from epidemiology, genetics, and biology. J Lipid Res.

[4] Burgess S (2018). Association of LPA variants with risk of coronary disease and the implications for lipoprotein(a)-lowering therapies: a mendelian randomization analysis. JAMA Cardiol.

[5] McConnell JP, Guadagno PA, Dayspring TD, Hoefner DM, Thiselton DL, Warnick GR, Harris WS (2014). Lipoprotein(a) mass: a massively misunderstood metric. J Clin Lipidol.

[6] Varvel S, McConnell JP, Tsimikas S (2016). Prevalence of elevated Lp(a) mass levels and patient thresholds in 532 359 patients in the United States. Arterioscler Thromb Vasc Biol.

[7] Tsimikas S (2020). Lipoprotein(a) reduction in persons with cardiovascular disease. N Engl J Med.

[8] Tsimikas S (2015). Antisense therapy targeting apolipoprotein(a): a randomised, double-blind, placebo-controlled phase 1 study. Lancet.

[9] Helgadottir A (2012). Apolipoprotein(a) genetic sequence variants associated with systemic atherosclerosis and coronary atherosclerotic burden but not with venous thromboembolism. J Am Coll Cardiol.

[10] Barre DE (1998). Lipoprotein (a) reduces platelet aggregation via apo(a)-mediated decreases in thromboxane A(2)production. Platelets.

[11] Barre DE (2003). Apolipoprotein (a) mediates the lipoprotein (a)-induced biphasic shift in human platelet cyclic AMP. Thromb Res.

[12] Barre DE (2007). Arginyl-glycyl-aspartyl (RGD) epitope of human apolipoprotein (a) inhibits platelet aggregation by antagonizing the IIb subunit of the fibrinogen (GPIIb/IIIa) receptor. Thromb Res.

[13] Blencowe C, Hermetter A, Kostner GM, Deigner HP (1995). Enhanced association of platelet-activating factor acetylhydrolase with lipoprotein (a) in comparison with low density lipoprotein. J Biol Chem.

[14] Miles LA, Plow EF (1985). Binding and activation of plasminogen on the platelet surface. J Biol Chem.

[15] Rand ML, Sangrar W, Hancock MA, Taylor DM, Marcovina SM, Packham MA, Koschinsky ML (1998). Apolipoprotein(a) enhances platelet responses to the thrombin receptor-activating peptide SFLLRN. Arterioscler Thromb Vasc Biol.

[16] Tsironis LD, Mitsios JV, Milionis HJ, Elisaf M, Tselepis AD (2004). Effect of lipoprotein (a) on platelet activation induced by platelet-activating factor: role of apolipoprotein (a) and endogenous PAF-acetylhydrolase. Cardiovasc Res.

[17] Berliner JA, Leitinger N, Tsimikas S (2009). The role of oxidized phospholipids in atherosclerosis. J Lipid Res.

[18] Barre DE (2004). Apoprotein (A) antagonises THE GPIIB/IIIA receptor on collagen and adp-stimulated human platelets. Front Biosci.

[19] Gries A, Gries M, Wurm H, Kenner T, Ijsseldijk M, Sixma JJ, Kostner GM (1996). Lipoprotein(a) inhibits collagen-induced aggregation of thrombocytes. Arterioscler Thromb Vasc Biol.

[20] Ezratty A, Simon DI, Loscalzo J (1993). Lipoprotein(a) binds to human platelets and attenuates plasminogen binding and activation. Biochemistry.

[21] Martinez C, Rivera J, Loyau S, Corral J, Gonzalez-Conejero R, Lozano ML, Vicente V, Angles-Cano E (2001). Binding of recombinant apolipoprotein(a) to human platelets and effect on platelet aggregation. Thromb Haemost.

[22] Malle E, Ibovnik A, Stienmetz A, Kostner GM, Sattler W (1994). Identification of glycoprotein IIb as the lipoprotein(a)-binding protein on platelets. Lipoprotein(a) binding is independent of an arginyl-glycyl-aspartate tripeptide located in apolipoprotein(a). Arterioscler Thromb.

[23] Hochholzer W (2006). Impact of the degree of peri-interventional platelet inhibition after loading with clopidogrel on early clinical outcome of elective coronary stent placement. J Am Coll Cardiol.

[24] Hochholzer W, Trenk D, Frundi D, Blanke P, Fischer B, Andris K, Bestehorn HP, Buttner HJ, Neumann FJ (2005). Time dependence of platelet inhibition after a 600-mg loading dose of clopidogrel in a large, unselected cohort of candidates for percutaneous coronary intervention. Circulation.

[25] Hochholzer W, Trenk D, Frundi D, Neumann FJ (2007). Whole blood aggregometry for evaluation of the antiplatelet effects of clopidogrel. Thromb Res.

[26] Boffa MB, Marar TT, Yeang C, Viney NJ, Xia S, Witztum JL, Koschinsky ML, Tsimikas S (2019). Potent reduction of plasma lipoprotein (a) with an antisense oligonucleotide in human subjects does not affect ex vivo fibrinolysis. J Lipid Res.

[27] Byun YS, Lee JH, Arsenault BJ, Yang X, Bao W, DeMicco D, Laskey R, Witztum JL, Tsimikas S (2015). Relationship of oxidized phospholipids on apolipoprotein B-100 to cardiovascular outcomes in patients treated with intensive versus moderate atorvastatin therapy: the TNT trial. J Am Coll Cardiol.

[28] Salsoso R (2020). Relation of high lipoprotein (a) concentrations to platelet reactivity in individuals with and without coronary artery disease. Adv Ther.

[29] O’Donoghue ML (2019). Lipoprotein(a), PCSK9 inhibition, and cardiovascular risk. Circulation.

[30] Bittner VA (2020). Effect of alirocumab on lipoprotein(a) and cardiovascular risk after acute coronary syndrome. J Am Coll Cardiol.

[31] Tsimikas S, Gordts P, Nora C, Yeang C, Witztum JL (2020). Statin therapy increases lipoprotein(a) levels. Eur Heart J.

