# Supplementary material for: Association of lipoprotein(a) with intrinsic and on-clopidogrel platelet reactivity
Source: J Thromb Thrombolysis. 2021 Jul 2;53(1):1–9. doi: 10.1007/s11239-021-02515-2 (PMC8791920; doi:10.1007/s11239-021-02515-2)
Supplement: Supplementary file 1 — Supplementary file1 (DOCX 179 kb) [file 11239_2021_2515_MOESM1_ESM.docx]

**Supplemental figure 1: Kaplan-Meier-Curve for death or myocardial infarction according to quartiles of Lp(a) levels**

Shown are the Kaplan-Meier-Curves for death or myocardial infarction stratified to quartiles of Lp(a).

**Supplemental figure 2: Light transmission aggregometry.**


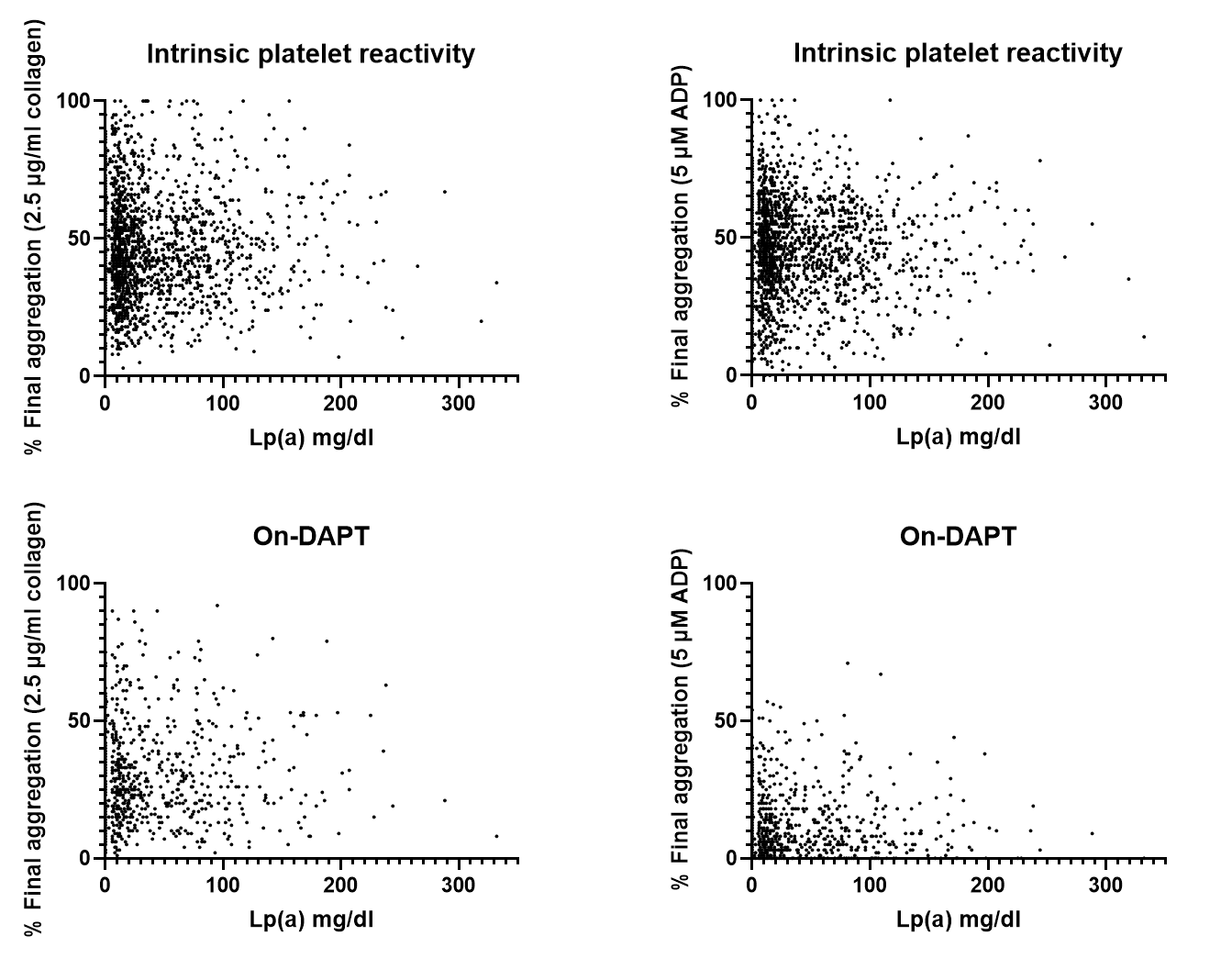


Shown is the intrinsic and on-dual antiplatelet therapy (DAPT) platelet reactivity tested 24h following loading with clopidogrel**.**

**Supplemental figure 3: Surface protein expression.**


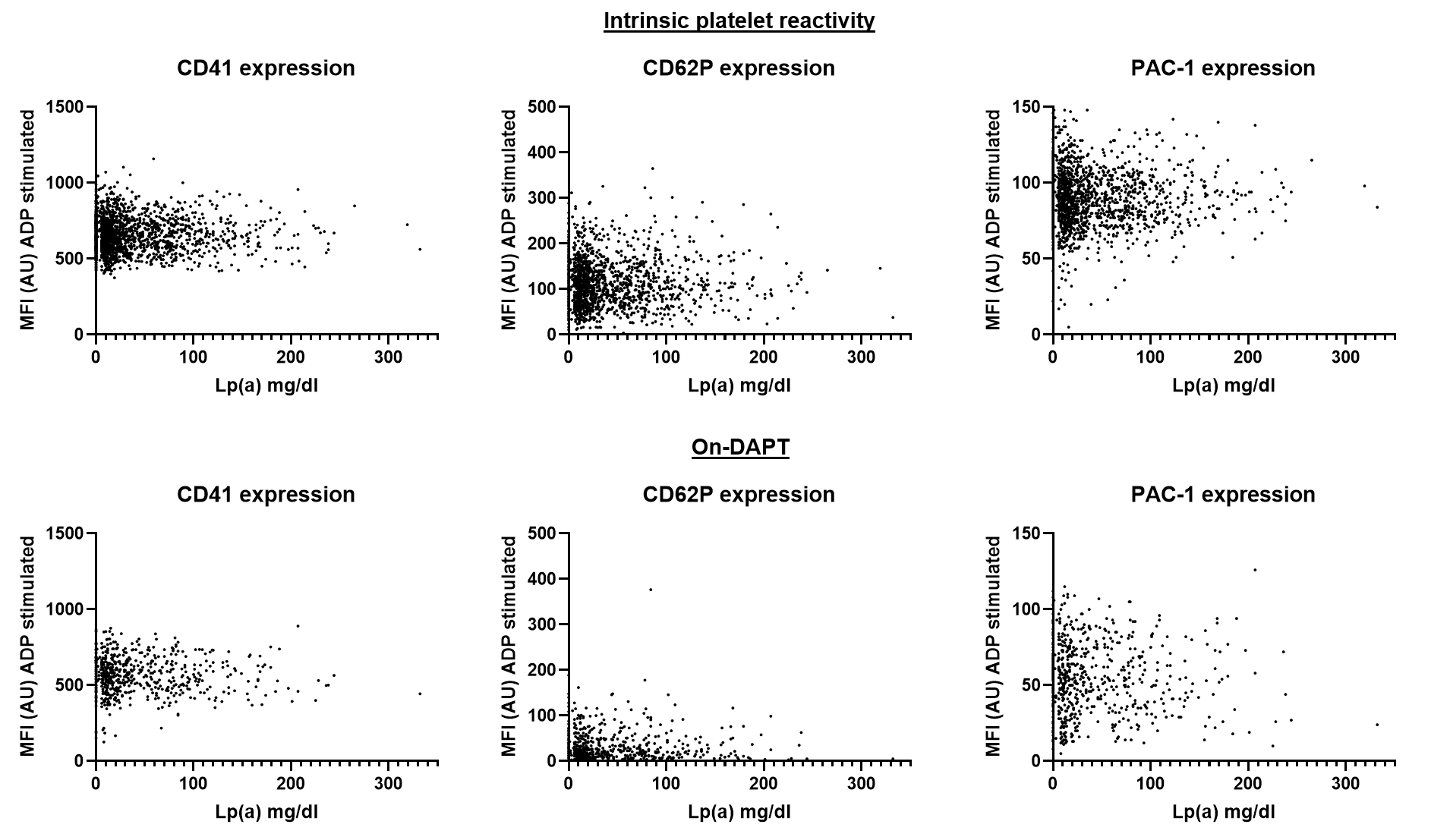


Shown is the intrinsic and on- dual antiplatelet therapy (DAPT) platelet reactivity tested 24h following loading with clopidogrel**.**
